# A Reply to Dr. Talbot

**Published:** 1899-02

**Authors:** C. DeW. Lukens

**Affiliations:** St. Louis, Mo.


### Domestic Correspondence.

### A REPLY TO DR. TALBOT.

To the Editor:

Sir,— In the International Dental Journal for October, 1898, appears an article under the head of "Double Resection of the Lower Maxilla," by Dr. Eugene S. Talbot, questioning the priority and criticising the technique of this operation as published by Dr. Edward H. Angle in the *Dental Cosmos* for August, 1898.

He says, "The late Dr. W. W. Allport, who suggested this operation over four decades ago, consulted the late Dr. Brainard, of Chicago, admittedly then one of the most skilful American surgeons, in regard to its propriety." As the late Dr. Allport was a man who contributed liberally to the literature of his profession, it is only in justice to him that the chapter and paragraph be given in which he refers to this operation. As modern men of science claim and are credited with priority from date of publication, it seems to savor of degeneracy to make claims on hearsay evidence of forty years ago. Through the pursuance of such childish methods worthy men are cheated of their reward and the seekers after truth unable to give credit where it is justly due. Further, it is surprising that Dr. Talbot should be willing to stake his reputation by assuming to diagnose and suggest treatment for cases which he has never seen. He remarks that in the operation as suggested by Dr. Allport the section was to be "cut in such a manner as to bring the incisors directly in contact with the superior incisors, and not throw the anterior part of the jaw downward, as would be the case in Dr. Angle's operation."

When we consider that in this particular case the inferior incisors projected to a point well above the labio-gingival margin of the superior incisors, it would seem that even a school-boy's conception of the most simple laws of mechanics would indicate that wedge-shaped pieces of bone must be removed. Dr. Talbot finally closes his article with one of those sweeping and dogmatic statements, so well known to all who are familiar with his writings and always resorted to in his efforts to convince and impress in the absence of facts or accurate data. He says, "The deformity is the result of arrest of development of the face and superior maxilla rather than an excessive development of the lower jaw." As Dr. Talbot has stood on a street corner of London and in an hour or so measured the faces of twenty thousand people, it is only reasonable to suppose that he unquestionably possesses the ability to note deviations from the normal facial line. But it would seem that in this case, where the superior maxilla was well up to the average in size and contains a full complement of large, well-developed teeth symmetrically arranged, that it would present a good argument in favor of the excessive development of the inferior maxilla, which is further borne out by the fact that in this case the lower maxilla *was* enormously over-developed, and would be easily detected from the engravings even by a freshman student.

A theory sometimes blinds the man who holds it, and not only hinders his own progress, but makes him a stumbling-block for others. "The difference between a horse and a hobby," said the lunatic, "is that one can get off a horse." Lovers of science are men of broad vision and generous enough to give honor where honor is due, and too devoted to their art to hinder its progress in the least degree.

When conservatism and opinionism have developed into bigotry, a man will be found too dull to see the truth and too narrow to acknowledge it.

Yours respectfully,
C. DeW. Lukens.

St. Louis, Mo.

[The foregoing letter is published at the request of the writer, and with the hope that the fact of priority may be established. The spirit in which this reply is evidently written is not to be commended, as it tends to defeat its own object.—Ed.]

